# Antileishmanial Activity of Warifteine: A Bisbenzylisoquinoline Alkaloid Isolated from *Cissampelos sympodialis* Eichl. (Menispermaceae)

**DOI:** 10.1100/2012/516408

**Published:** 2012-09-02

**Authors:** Eliete Cavalcanti da Silva, Cynthia Dias Rayol, Paloma Lys Medeiros, Regina Célia Bressan Queiroz Figueiredo, Márcia Regina Piuvezan, José Maria Brabosa-Filho, Alexsandro Fernandes Marinho, Teresinha Gonçalves Silva, Gardenia Carmen Gadelha Militão, Ana Paula Pimentel Cassilhas, Paulo Paes de Andrade

**Affiliations:** ^1^Departamento de Histologia e Embriologia, Centro de Ciências Biológicas, Universidade Federal de Pernambuco, Avenida Professor Moraes Rego 1265, Cidade Universitária, 50670-901 Recife, PE, Brazil; ^2^Departamento de Microbiologia, Centro de Pesquisas Aggeu Magalhães/FIOCRUZ, 50670-420 Recife, PE, Brazil; ^3^Laboratório de Tecnologia Farmacêutica, Universidade Federal da Paraíba, Campus I, 58051-900 João Pessoa, PB, Brazil; ^4^Departamento de Antibióticos, Centro de Ciências Biológicas, Universidade Federal de Pernambuco, Avenida Professor Moraes Rego, 1265, Cidade Universitária, 50670-901 Recife, PE, Brazil; ^5^Departamento de Ensino de Enfermagem, Universidade Salgado de Oliveira, 51170-000 Recife, PE, Brazil; ^6^Departamento de Genética, Centro de Ciências Biológicas, Universidade Federal de Pernambuco, Avenida Professor Moraes Rego 1265, Cidade Universitária, 50670-901 Recife, PE, Brazil

## Abstract

*Leishmania (L.) chagasi* is the etiological agent of visceral leishmaniasis, an important endemic zoonosis in the American continent, as well as in many other countries in Asia, Africa, and Mediterranean Europe. The treatment is difficult due to the high toxicity of the available drugs, high costs, and emergence of resistance in the parasites. Therefore, there is an urgent need for new leishmanicidal agents. The bisbenzylisoquinoline alkaloids have been related to antibacterial, antiprotozoal, and antifungal activities. The aim of this study was to evaluate the growth inhibitory activity of warifteine (bisbenzylisoquinoline alkaloid) against *L. chagasi* promastigotes in axenic cultures and the occurrence of drug-induced ultrastructural changes in the parasite. This bisbenzylisoquinoline alkaloid was isolated from the leaves and roots of *Cissampelos sympodialis* Eichl. (Menispermaceae), a plant commonly used for the treatment of various diseases in Brazilian folk medicine. Using the purified warifteine, the 50% inhibitory concentration (IC_50_) was determined at 0.08 mg/mL after 72 h in culture, inducing significant changes in the parasite morphology, like aberrant multisepted forms and blebs in the plasma membrane. In conclusion, warifteine represents an attractive candidate for future pharmacological studies aiming new leishmanicidal drugs.

## 1. Introduction

*Leishmania (L.) chagasi*, a trypanosomatid parasite, is the etiological agent of visceral leishmaniasis (VL) in the American continent and it is now admitted to be the same species causing visceral leishmaniasis in Europe and certain parts of Africa (*L.(L.) infantum*). The disease is prevalent in more than 80 countries in Asia, Africa, the Americas, and Mediterranean Europe [1]. When not treated, death is expected to occur after a period of 4 months to one year [2]. 

 The therapeutic options currently available have serious limitations, such as the emergence of parasite resistance and high toxicity [2, 3]. The aqueous infusion of *Cissampelos sympodialis* Eichl. (Menispermaceae), popularly known in Brazil as “milona,” is widely used in folk medicine to treat asthma, bronchitis, and rheumatism [4]. The genus *Cissampelos* is rich in bisbenzylisoquinoline alkaloids which are known to have various pharmacological properties including antiparasitic activity, in particular against *Leishmania *sp. [5], *Trypanosoma cruzi* [6], and *Plasmodium *sp. [7, 8].

Considering that the bisbenzylisoquinoline alkaloids have been shown to display prominent antibacterial, as well as antiprotozoal and antifungal activities [5, 9, 10], we proposed to evaluate the growth inhibitory activity of warifteine against *L. chagasi* promastigotes in axenic cultures and report its inhibitory action *in vitro* and the occurrence of ultrastructural changes.

## 2. Materials and Methods

### 2.1. Extraction and Isolation of Warifteine

Warifteine was purified from leaves of *Cissampelos sympodialis* Eichl. (Menispermaceae) grown at the Botanical Garden of the Laboratório de Tecnologia Farmacêutica/Universidade Federal da Paraíba/UFPB/João Pessoa, Brazil (voucher specimen Agra-1456). The leaves of *C. sympodialis* were dried at 50°C in an oven and pulverized and the powder extracted with 70% ethanol in water at 70°C for 5 days. The plant hydroalcoholic extract was submitted to procedures aimed to isolate the alkaloids, using column and thin-layer chromatography (TLC). The plant hydroalcoholic extract was dissolved in 3% HCl and extracted several times with CHCl_3_. The aqueous fraction was basified with NH_4_OH to pH 9 and again extracted with CHCl_3_. The CHCl_3_ extract was washed with H_2_O, dried (MgSO_4_), and the solvent evaporated to afford the total tertiary alkaloid fraction (TTA). The TTA was subjected to chromatography column over alumina, eluting with hexane containing increasing amounts of CHCl_3_, CHCl_3_ with increasing amounts of MeOH and finally with MeOH. The fraction eluted with CHCl_3_–MeOH (49 : 1), after further purification by TLC (1.0 mm layer), yielded the isolation of the bisbenzylisoquinoline alkaloid warifteine (0.031%). The identification of the warifteine was performed by analyzing ^1^H and ^13^C NMR spectral data compared with those published in the literature. Warifteine was endotoxin free and it had 100% purity, as determined by NMR and mass spectroscopy [11, 12].

### 2.2. Parasites Isolation and Culture

The strain of *L. chagasi* used in this study was isolated from an axenic culture of bone marrow aspirate of a dog with visceral leishmaniasis originated from Patos (PB, Brazil) and its taxonomic identification was confirmed by isoenzyme profiling and PCR using specific primers directed to DNA minicircles as previously described [13]. The promastigotes were routinely grown in Liver Infusion Tryptose medium (LIT, HiMedia, Laboratories Pvt. Ltda., Mumbai, India) at 26°C, supplemented with 10% heat-inactivated fetal bovine serum (FBS) (LGC Biotechnology Ltda., Brazil), 0.1% penicillin and streptomycin, and 0.2% hemin (Sigma Chemical Co., St. Louis, MO, USA). 

### 2.3. Antileishmanial Activity *In Vitro*

The promastigotes were seeded in 24-well culture microplates (CORNING Costar, Corning Incorporated, NY, USA) at an initial concentration of 2 × 10^6^ cells/mL. Meglumine antimonite and warifteine were diluted in LIT supplemented with 0.5% dimethyl sulfoxide (LIT-DMSO) and added to the wells at different concentrations. The control group consisted of promastigotes grown on LIT-DMSO (Sigma Chemical Co., St. Louis, MO, USA) only. Drug concentrations ranged from 2.5 to 5 mg/mL for meglumine antimonite and from 0.05 to 0.15 mg/mL for warifteine. Cell growth assessment was carried out by cell counting in a Neubauer chamber at 24 h intervals, 24, 48, and 72 h after incubation. Relative growth from the last sampling time was used to calculate the IC_50_ (concentration that inhibits growth by 50%). All the experiments were performed in triplicate.

### 2.4. Cytotoxicity Test

Warifteine cytotoxicity was evaluated against human laryngeal cancer cells (HEP-2 cells) and human lung mucoepidermoid (NCI H-292) cells, both provided by the Rio de Janeiro Cell Bank (BCRJ). They were grown in DMEM medium supplemented with 10% calf serum at a concentration of 1 × 10^5^ cells/mL, at 37°C, 5% CO_2_. Cell viability was determined using MTT assay at 595 nm [14]. All experiments were performed in triplicate.

### 2.5. Scanning Electron Microscopy

To evaluate parasite ultrastructural alterations by scanning electron microscopy, *L. chagasi* promastigotes were grown for 72 h as described in LIT-DMSO or the same medium containing 80 *μ*g/mL warifteine; they were subsequently collected by centrifugation at 1500 ×g, washed with 0.1 M phosphate buffer (pH 7.2) and fixed in 2.5% glutaraldehyde, 4% paraformaldehyde in 0.1 M phosphate buffer. After washing twice in the same buffer, the parasites were adhered to glass slides previously coated with 0.1% aqueous poly-I-lysine for 30 min at 37°C. Subsequently, the slides were washed twice with 0.1 M phosphate buffer, postfixed in solution of 1% OsO_4_ for 1 h at room temperature, and washed twice again with 0.1 M phosphate buffer. All samples were dehydrated in a graded series of ethanol (30–100%), critical point dried using CO_2_, mounted on metal stubs, and coated with gold (5–30 nm) for observation in a scanning electron microscope (JEOL T-200). 

### 2.6. Statistical Analysis

 The results were expressed as mean values ± standard deviation (S.D.). Statistical analysis was made by Kruskal-Wallis test and *P* values < 0.05 were considered significant.

## 3. Results

**Figure 1:**
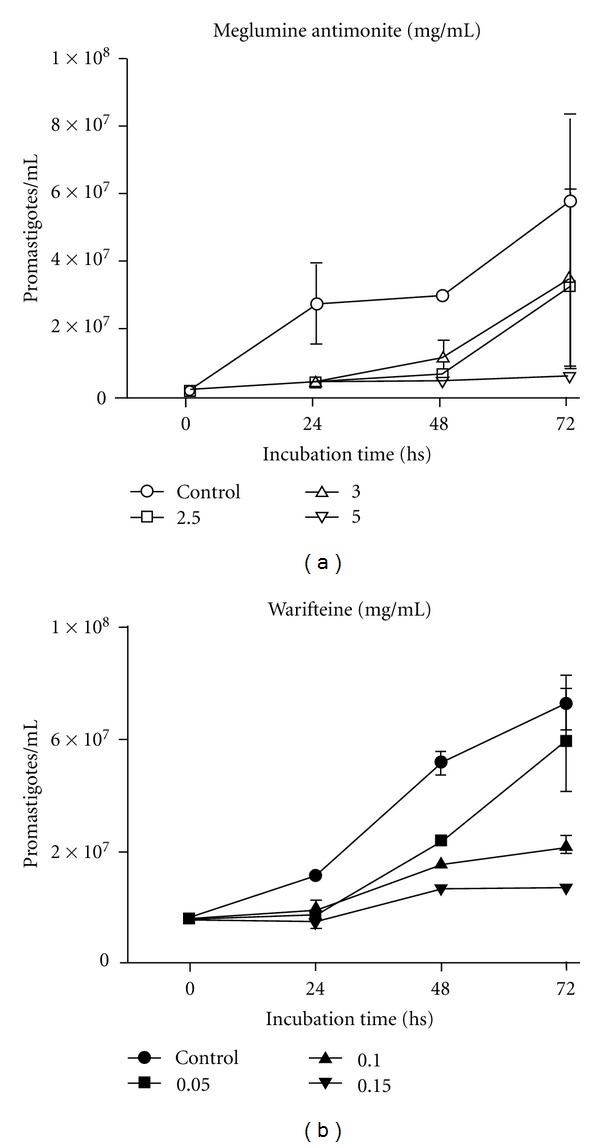
*In vitro* effects of different concentrations of meglumine antimonite (a) and warifteine (b) on the growth kinetics of *L. chagasi *promastigotes forms. Results are expressed as the mean of triplicate experiments.

Figure 1 shows the *in vitro* effects of different concentrations of meglumine antimonite and warifteine on the growth of *L. chagasi* promastigotes. Growth inhibition was directly proportional to meglumine antimonite and warifteine concentrations. Inhibition reached 89.3% for meglumine antimonite at 5 mg/mL and 70% for warifteine at 0.15 mg/mL. Warifteine antileishmanial activity was estimated by the IC_50_ concentration at 72 h after incubation. Warifteine was found to exhibit a higher inhibitory activity against *L. chagasi* (IC_50_ = 0.08 mg/mL = 135 *μ*M) than the reference drug meglumine antimonite (IC_50_ = 2.5 mg/mL). 

The cytotoxicity assay resulted in an IC_50_ of 0.056 ± 0.0026 mg/mL (NCI-H292) and of 0.067 ± 0.0016 mg/mL (HEp-2).

**Figure 2:**
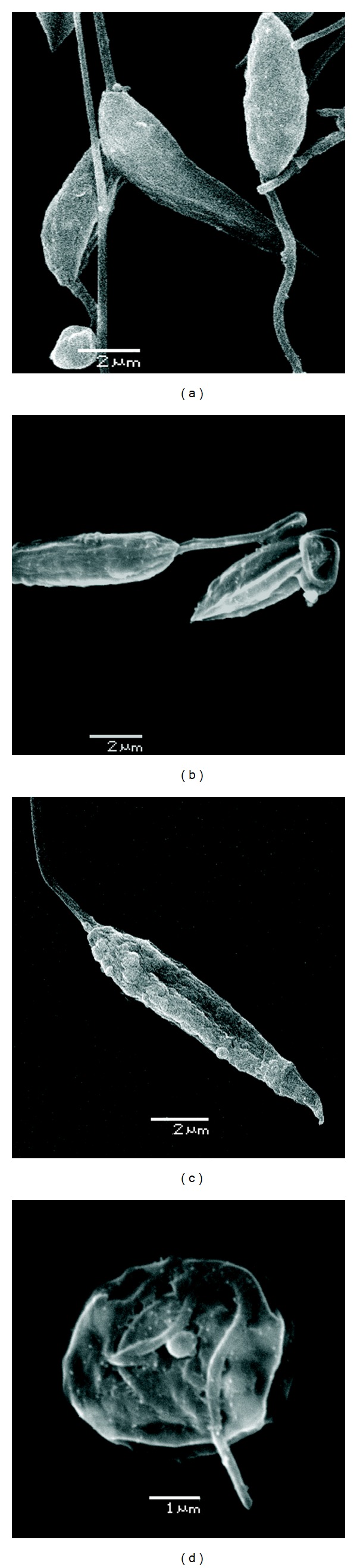
In (a) scanning electronmicrograph (SEM) of control *L. chagasi*, displaying the characteristic morphology of promastigotes. In (b), (c), and (d) SEM of warifteine-treated parasites, showing septa (b) “blebs” scattered over the plasma membrane (c) and rounded shape (d). Magnification 6.500 (a), 7.000 (b), 7.500 (c), and 13.000 (d).

 The analysis of scanning electronmicrographs of treated parasites demonstrated that warifteine affected the parasite surface. Some parasites lost their characteristic elongated shape and presented a round shape and frequently also longitudinal septa, as well as blebs scattered over their plasma membrane. The blebs were usually semispherical, but their sizes, number and location varied considerably (Figure 2). No ultrastructural change was observed in promastigotes grown with LIT-DMSO for 72 h, showing the elongated normal morphology. 

## 4. Discussion

We demonstrated for the first time that warifteine, a bisbenzylisoquinoline alkaloid, isolated from *Cissampelos sympodialis* Eichl., inhibited the growth of *L. chagasi* promastigotes *in vitro*. The inhibition was stronger than that observed with a pentavalent antimonial, as the effective concentration for warifteine was more than 30 times smaller (0.15 mg/mL versus 5 mg/mL). The drug was also effective *in vitro* against *Leishmania amazonensis*, with a IC_50_ = 4.3 *μ*g/mL [15]. There are no other reports on the leishmanicidal activity of this drug, except for extracts from the same plant. However, two other alkaloids are also present in leaf extracts, methylwarifteine, and milonine [16], and therefore it is not possible to quantitatively compare inhibitory concentrations. Nevertheless, the warifteine IC_50_ value is similar to that of the trivalent antimonial, which ranges from 5.5 to 30.2 mg/mL depending on the *L. infantum* strain [17].

The previously reported spasmolytic action of warifteine was related to the relaxation of smooth muscle, due to the inhibition of Ca^2+^ channels and alteration of intracellular Ca^2+^ stores sensitive to noradrenaline. Moreover, the ethanolic extract from its root and leaves increases cAMP levels in tracheal smooth muscle cells through the inhibition of the cAMP degrading enzyme phosphodiesterase [4, 11]. These reports are in agreement with the present results, demonstrating the action of warifteine on the parasite surface, and suggest that the growth inhibition of *L. chagasi in vitro* may be related to these structural changes, through the decrease in intracellular calcium concentration and the consequent change in the structure of subpellicular microtubules and other filaments immediately below the plasma membrane; in fact, warifteine is reported to change intracellular calcium concentrations [11]. A similar picture (blebs) was observed after incubation of *L. chagasi* with nimodipine, a calcium channel blocker with an action similar to warifteine [18]. Besides being directly leishmanicidal, warifteine could control parasite load *in vivo* through the enhancement of NO production [19]. Actually, Costa and colleagues [12] reported the NO increase in macrophages induced by warifteine. 

Besides its leishmanicidal activity, warifteine shows moderate cytotoxicity against cell lineages isolated from mammals (HEp-2 and NCI-H292). In a previous report, warifteine was found to be more toxic than milonine, but still within levels that would not preclude further therapeutic assays [20]. 

In conclusion, due to its inhibitory effect and induction of sharp ultrastructural changes on *Leishmania chagasi* cell surface, warifteine is a potential antileishmanial candidate for future evaluation in pharmacological studies *in vivo.*
